# Metabolic Effects and Safety Aspects of Acute D-allulose and Erythritol Administration in Healthy Subjects

**DOI:** 10.3390/nu15020458

**Published:** 2023-01-15

**Authors:** Fabienne Teysseire, Valentine Bordier, Aleksandra Budzinska, Lukas Van Oudenhove, Nathalie Weltens, Christoph Beglinger, Bettina K. Wölnerhanssen, Anne Christin Meyer-Gerspach

**Affiliations:** 1 St. Clara Research Ltd., St. Claraspital, 4002 Basel, Switzerland; 2 Faculty of Medicine, University of Basel, 4001 Basel, Switzerland; 3 Laboratory for Brain-Gut Axis Studies, Translational Research Center for Gastrointestinal Disorders, Department of Chronic Diseases and Metabolism, KU Leuven, 3000 Leuven, Belgium; 4 Leuven Brain Institute, Katholieke Universiteit Leuven, 3000 Leuven, Belgium; 5 Cognitive and Affective Neuroscience Laboratory, Department of Psychological and Brain Sciences, Dartmouth College, Hanover, NH 03755, USA

**Keywords:** D-allulose, erythritol, sweeteners, glycemic control, ghrelin, blood lipids, uric acid, hsCRP, healthy subjects

## Abstract

The rapid increase in sugar consumption is associated with various negative metabolic and inflammatory effects; therefore, alternative sweeteners become of interest. The aim of this study was to investigate the metabolic effects and safety aspects of acute D-allulose and erythritol on glucose, insulin, ghrelin, blood lipids, uric acid, and high-sensitive C-reactive protein (hsCRP). In three study visits, 18 healthy subjects received an intragastric administration of 25 g D-allulose or 50 g erythritol, or 300 mL tap water (placebo) in a randomized, double-blind and crossover order. To measure the aforementioned parameters, blood samples were drawn at fixed time intervals. Glucose and insulin concentrations were lower after D-allulose compared to tap water (*p* = 0.001, d_z_ = 0.91 and *p* = 0.005, d_z_ = 0.58, respectively); however, Bayesian models show no difference for insulin in response to D-allulose compared to tap water, and there was no effect after erythritol. An exploratory analysis showed that ghrelin concentrations were reduced after erythritol compared to tap water (*p* = 0.026, d_z_ = 0.59), with no effect after D-allulose; in addition, both sweeteners had no effect on blood lipids, uric acid and hsCRP. This combination of properties identifies both sweeteners as excellent candidates for effective and safe sugar alternatives.

## 1. Introduction

Fructose is typically found in fruits, sucrose, honey and high fructose corn syrup (HFCS). The excessive consumption of foods and beverages containing HFCS or sucrose are, however, associated with various risk factors such as insulin resistance, elevated blood lipids and uric acid, as well as an increase in systemic inflammation [1,2,3,4]. These negative metabolic effects lead to an increased risk of non-communicable diseases such as obesity, diabetes mellitus type 2 (T2DM), cardiovascular diseases (CVD) and hyperuricemia [5,6,7,8]. From a preventive perspective, HFCS and sucrose consumption should be reduced; therefore, there is growing interest in the use of efficacious and safe alternative sweeteners.

D-allulose and erythritol, two naturally occurring sweeteners, are interesting alternatives. Both sweeteners have a sweetness of around 60–80% relative to sucrose and are associated with several positive health effects.

It was shown that D-allulose, a C_3_ epimer of D-fructose, does not affect blood glucose in response to an oral glucose tolerance test (OGTT) in healthy humans [9]. More importantly, Franchi et al. [10] have reported that an acute intake of D-allulose in combination with 50 g of sucrose leads to a dose-dependent reduction in glucose and insulin concentrations compared to sucrose alone. In addition, several studies have found that D-allulose reduces postprandial blood glucose concentrations compared to either maltodextrin, a tea without D-allulose, or fructose in healthy participants and participants with prediabetes as well as T2DM [11,12,13]. Similar to fructose, D-allulose stimulates the release of gastrointestinal (GI) satiation hormones such as cholecystokinin (CCK), glucagon-like peptide-1 (GLP-1), and peptide tyrosine tyrosine (PYY), thereby modulating appetite [14,15,16]. In mice, it was shown that a central injection of D-allulose inhibited ghrelin-responsive neurons in the arcuate nucleus (ARC) in the hypothalamus [17]. Whether D-allulose affects orexigenic ghrelin concentrations in humans is currently unknown. Furthermore, it was shown that D-allulose, compared to sucralose, reduces body mass index (BMI) including abdominal and subcutaneous fat areas in a 12─week trial including participants that are overweight and participants with obesity, with no adverse effect on blood lipids [18]. Administering D-allulose to patients with high low-density lipoprotein (LDL) cholesterol levels for 48 weeks did not increase blood lipids or high-sensitive C-reactive protein (hsCRP) [19]. Finally, daily intake of D-allulose in tea with a standard meal over 12 weeks did not affect uric acid concentrations [12].

Whether an acute intragastric administration of 25 g of pure D-allulose is efficacious and safe in regulating glucose, insulin and ghrelin concentrations, as well as blood lipids, uric acid and hsCRP has not been investigated.

Erythritol, for its part, is a four-carbon sugar alcohol with the formula C_4_H_10_O_4_ and occurs naturally in fruits, vegetables and fermented food and drinks [20]. Erythritol does not affect glucose and insulin concentrations and seems to have protective effects on endothelial function in patients with T2DM [21,22,23,24]. Although erythritol provides zero calories, it induces the release of CCK, GLP-1 and PYY that is similar to glucose and sucrose [22,23]. A recent study indicates that ghrelin concentrations are suppressed in response to oral erythritol in healthy participants [25]. In a pilot dose-ranging study, acute ingestion of erythritol did not affect blood lipid and uric acid concentrations [23]. Based on toxicological and safety data, erythritol is generally recognized as safe (GRAS) by the Food and Drug Administration (FDA) in the United States for its intended use in foods [26]. However, the effect of erythritol on hsCRP is not known.

The aim of this study is to investigate the metabolic effects of acute intragastric administration of 25 g D-allulose or 50 g erythritol on glucose, insulin and ghrelin concentrations as well as to assess safety aspects of both alternative sweeteners on blood lipids, uric acid and hsCRP concentrations. The rationale for the intragastric administration of the solutions was to bypass oro-sensory exposure. We hypothesized that glucose and insulin concentrations will be similar, and ghrelin will be reduced in response to D-allulose and erythritol compared to tap water, respectively.

## 2. Subjects and Methods

### 2.1. Approval

The Ethics Committee of Northwestern-and central Switzerland (EKNZ): 2019-01111 approved the trial. The trial was conducted in accordance with the current version of the Declaration of Helsinki, the guidelines of Good Clinical Practice (GCP) issued by the International Council on Harmonisation (ICH) and the Swiss law and Swiss regulatory authority’s requirements. All participants gave their informed consent for inclusion before inclusion in the study. The study was registered at ClinicalTrials.gov (NCT04027283).

### 2.2. Subjects

Twenty-one subjects were recruited via advertisement at the local university. Subjects were eligible for the study when meeting all of the subsequent inclusion criteria: age between 18−55 years, BMI of 19.0–24.9 kg/m^2^ and normal eating habits (no diets, no dietary changes). Exclusion criteria were medical or drug abuse including alcohol dependence, acute or chronic infection or illness, illnesses affecting the GI tract, pre-existing consumption of D-allulose and/or erythritol more than once a week, pregnancy and involvement in another study with an investigational drug within 30 days preceding and/or during the current study.

### 2.3. Design and Procedure

The study used a double-blind, placebo-controlled, cross-over design and was conducted between September 2019 and September 2020. Part of the results and the sample of this study were reported elsewhere [14]. Each subject took part in three separate study visits as follows: 25 g D-allulose, 50 g erythritol or 300 mL tap water (placebo). The solutions were dissolved in 300 mL tap water. The rationale for the doses was chosen for the primary outcome of this study (GI satiation hormone release) and was based on the following considerations as previously described [14]: 50 g erythritol induces CCK, GLP-1, and PYY release reliably without GI side effects and corresponds to around 33.5 g of sucrose typically found in sweet beverages [22]. The effect of D-allulose on GI satiation hormones was not investigated before in humans. The recommended maximal single dose–where no GI side effects are observed–is 25 g [27]. The order of the study visits was randomized and counterbalanced among subjects. The study visits took place at least three days apart and after a 10-h overnight fast. All study visits started at ~0830 in the morning and, upon arrival, a cannula was inserted into a forearm vein for blood collection. Next, a nasogastric feeding tube (external diameter of 8 French) was inserted into the stomach. The rationale for intragastric administration of the solutions was to bypass oro-sensory exposure (e.g., taste and intensity) directly affecting brain mechanisms that may influence physiological/endocrine responses [28,29].

After taking blood samples in a fasting state (t = −10 and −1 min), subjects received one of the solutions (at t = 0 min) via the nasogastric feeding tube over two minutes.

**Figure 1:**
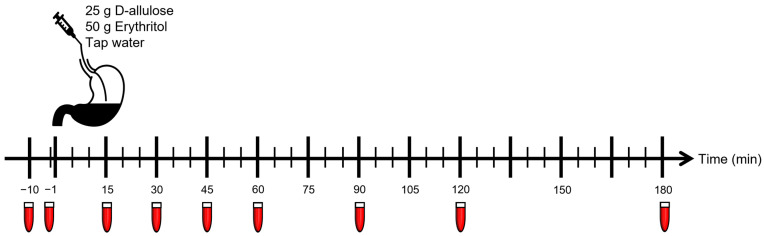
Study timeline: Intragastric administration of the solutions at t = 0 min to 18 healthy subjects in a randomized, double-blind, crossover order, in three different study visits after an overnight fast. The red tubes indicate blood sample collection.

More blood samples were taken at t = 15, 30, 45, 60, 90, 120 and 180 min for the analysis of glucose, insulin and ghrelin, and at t = 30, 60, and 120 min, for analysis of blood lipids, uric acid and hsCRP (Figure 1). Blood pressure and heart rate were measured at the beginning and at the end of each study visit. The subjects, including the personnel performing the study visits and blood analysis, were blinded regarding the content of the administered solutions. 

### 2.4. Blood Sample Collection and Processing

Blood samples for glucose, insulin and ghrelin were collected on ice into tubes containing EDTA (6 µmol/L blood) and a protease-inhibitor cocktail (complete, EDTA-free, 1 tablet/50 mL blood, Roche, Mannheim, Germany). Blood lipids, uric acid and hsCRP blood samples were collected on ice into serum tubes. After centrifugation (4 °C at 3000 rpm for 10 min), the samples were processed into different aliquots (for the ghrelin samples, 150 µL of 1N hydrochloric acid was added) and stored at −80 °C until analysis.

### 2.5. Materials

D-allulose was purchased from Tate & Lyle (Decatur, IL, USA) and erythritol from Mithana GmbH (Zimmerwald, Switzerland).

### 2.6. Laboratory Analysis

*Plasma glucose and insulin *were measured with an enzymatic assay from Beckman-Coulter and an electrochemiluminescence immunoassay (ECLIA) (Rothen Medizinische Laboratorien AG, Basel, Switzerland), respectively. The intra- and inter-assay variability is below 0.7% and 0.9% (glucose) and below 4.3% and 5.3% (insulin). The appropriate range of the assays are 0.6 to 45 mmol/L (glucose) and 0.4 to 1000 μU/mL (insulin). *Plasma octanoylated ghrelin *was measured by a radioimmunoassay with 125l [Tyr24] human ghrelin [1,2,3,4,5,6,7,8,9,10,11,12,13,14,15,16,17,18,19,20,21,22,23] as a tracer and a rabbit antibody against human ghrelin [1,2,3,4,5,6,7,8] (final dilution 15/100000), which does not cross-react with desoctanoylated ghrelin, as described previously in more detail [30].* Serum blood lipids, uric acid and hsCRP* were measured with enzymatic assays from Beckman-Coulter (Rothen Medizinische Laboratorien AG, Basel, Switzerland). The intra- and inter-assay variability is below 0.7% and 0.8% (cholesterol), below 2.26% and 2.71% (LDL), below 0.85% and 1.92% (HDL), below 1.06% and 1.76% (triglycerides), below 1.55% and 2.44% (uric acid) and below 5% and 7.5% (hsCRP). The appropriate range of the assays are 0.5 to 18.0 mmol/L (cholesterol), 0.3 to 10.3 mmol/L (LDL), 0.05 to 4.65 mmol/L (HDL), 0.1 to 11.3 mmol/L (triglycerides), 89 to 1785 μmol/L (uric acid) and 0.2 to 80.0 mg/L (hsCRP).

### 2.7. Statistics

The sample size calculation to detect a difference between GI hormones in response to both alternative sweeteners compared to tap water was previously reported [14]. For the metabolic effects (glucose, insulin and ghrelin) and safety aspects (blood lipids, uric acid and hsCRP) parameters, no sample size calculations were performed. However, in a sensitivity power calculation, the sample size of 18 participants yields 80% power to detect a medium effect size (Cohen’s d = 0.65) for the comparison of D-allulose and erythritol with tap water using a one-tailed paired t-test with Holm multiple testing correction (α = 0.0375). The one-tailed test is justified by the directional nature of our hypothesis regarding the effects on ghrelin (see below).

Statistical analysis was conducted using SAS 9.4 (SAS Institute, Cary, NC, USA). Data is presented as the mean ± SEM unless otherwise stated. A two-tailed *p*-value < 0.05 was considered significant and Cohen’s d_z_ for paired *t*-tests was presented for effect sizes. Kolmogorov-Smirnov testing and quantile-quantile plots were used to assess normality; for instance, if necessary, natural logarithmic transformations of the data were used to normalize distributions. The visit number was included to control for putative order effects in all models. The metabolic and safety outcome variables were analyzed using linear mixed models on changes from baseline (average of pre-infusion time point(s) for the metabolic parameters) and absolute values for the safety aspect parameters. “Solution” and “time” were included as within-subject independent variables in the models (including their main effects and the interaction). The metabolic outcome models controlled for baseline values. To follow up on significant main or interaction effects, planned contrast analyses were performed to test the specific hypotheses, with stepdown Bonferroni (Holm) correction for multiple testing.

To test the hypotheses that glucose and insulin concentrations, in response to D-allulose and erythritol, will be similar to tap water and that ghrelin will be reduced in response to D-allulose and erythritol compared to tap water, respectively, we compared the post-infusion glucose, insulin and ghrelin concentration changes from the baseline between tap water and D-allulose or erythritol. We did not formulate any a priori hypotheses about the safety outcomes.

Given our hypothesis about glucose and insulin concentrations being similar for the two solutions compared to tap water, we complemented our frequentist statistical analysis with Bayesian analyses in two complementary ways. *First*, we ran Bayesian equivalents of the abovementioned linear mixed model analyses for these two outcomes using SAS PROC BGLIMM with 10,000 burn-in samples followed by 100,000 Markov chains. A weakly informative normal prior (µ = 0, σ = 2) was used for the fixed effects coefficients, while an uninformative uniform prior with upper limit 1000 was used for the variance parameter of the covariance matrix for the random substance effect, to downplay the role of a relatively informative prior on the posterior distribution. Diagnostics (trace, autocorrelations and density plots and effective sample sizes) were used to confirm Markov chain convergence. *Second*, to the best of our knowledge, Bayes factors were not implemented in the context of the Bayesian linear mixed model analysis outside the context of Bayesian model selection [31], and we calculated Bayes factors in a one-way repeated measures ANOVA analysis with the AUC of glucose or insulin concentrations as the dependent variable and the solution as the sole independent variable as implemented in the JASP 0.16.4.0 software [32].

Since the AUC of total serum ghrelin was not significant in the study by Sorrentino et al. [25] between erythritol and aspartame consumption, but time points t = 20, 30, and 45 min after erythritol consumption were, we further explored the time points t = 30 and 45 min of ghrelin concentrations in response to D-allulose and erythritol, compared to tap water, in the current study.

## 3. Results

Twenty-one subjects were randomized. There were three drop-outs (one subject withdrew due to knee surgery and two withdrew for personal reasons). A total of 18 subjects (5 males and 13 females, mean ± SD (range), age: 24 ± 4 (19–35) years and BMI 21.9 ± 1.7 (19.1–24.3) kg/m^2^ completed the three study visits. Complete data sets from all 18 subjects were available for analysis.

### 3.1. Plasma Glucose

**Figure 2:**
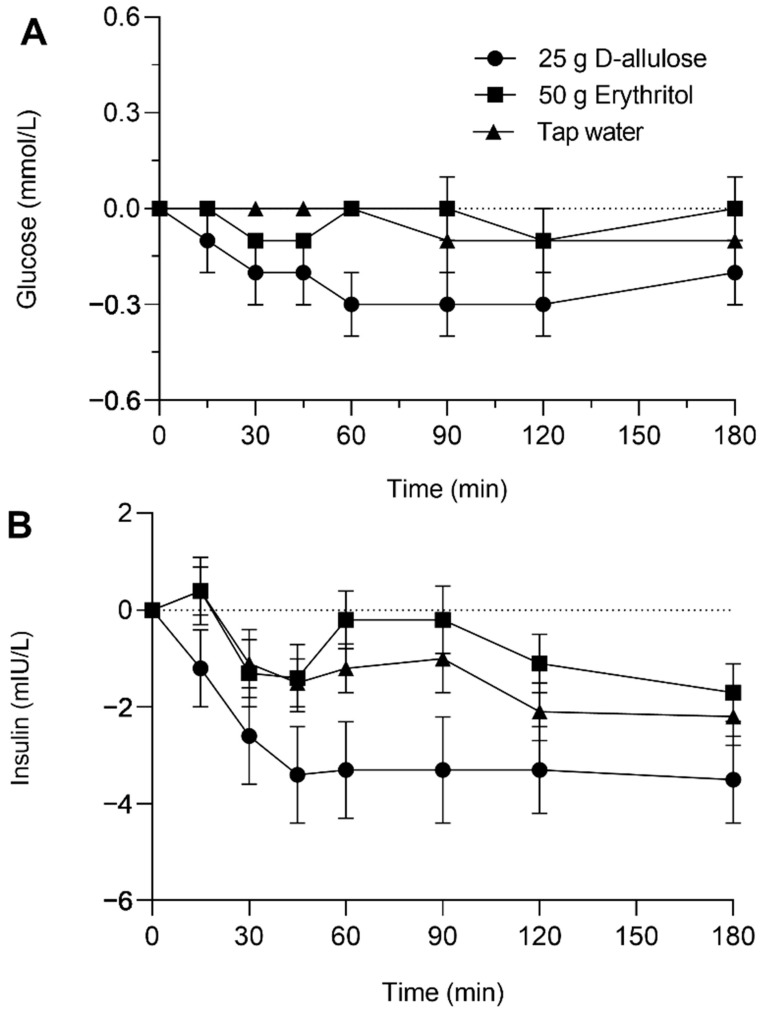
Glucose (**A**) and insulin (**B**) concentrations in response to intragastric administration of solutions containing 25 g D-allulose, 50 g erythritol or tap water to 18 healthy subjects. Data are expressed as mean ± SEM, and changes from baseline values are shown.

D-allulose decreased plasma glucose, whereas erythritol had no effect compared to tap water (Figure 2A). The main effect of the solution and the solution-by-time interaction effect were significant ((F (2, 41) = 8.86, *p* = 0.001) and (F (12, 166) = 3.20, *p* = 0.0004), respectively). Planned contrast analyses show that plasma glucose was lower after D-allulose vs. tap water, but not after erythritol vs. tap water (*p* = 0.001, d_z_ = 0.91 and *p* = 0.787, respectively). These results were corroborated by Bayesian linear mixed model analysis, showing a difference between D-allulose and tap water (estimate ± standard deviation (SD): −0.202 ± 0.078, highest probability density (HPD) interval −0.356 − −0.048), but not between erythritol and tap water (−0.018 ± 0.055, −95% HPD 0.12–0.097).

**Figure 3:**
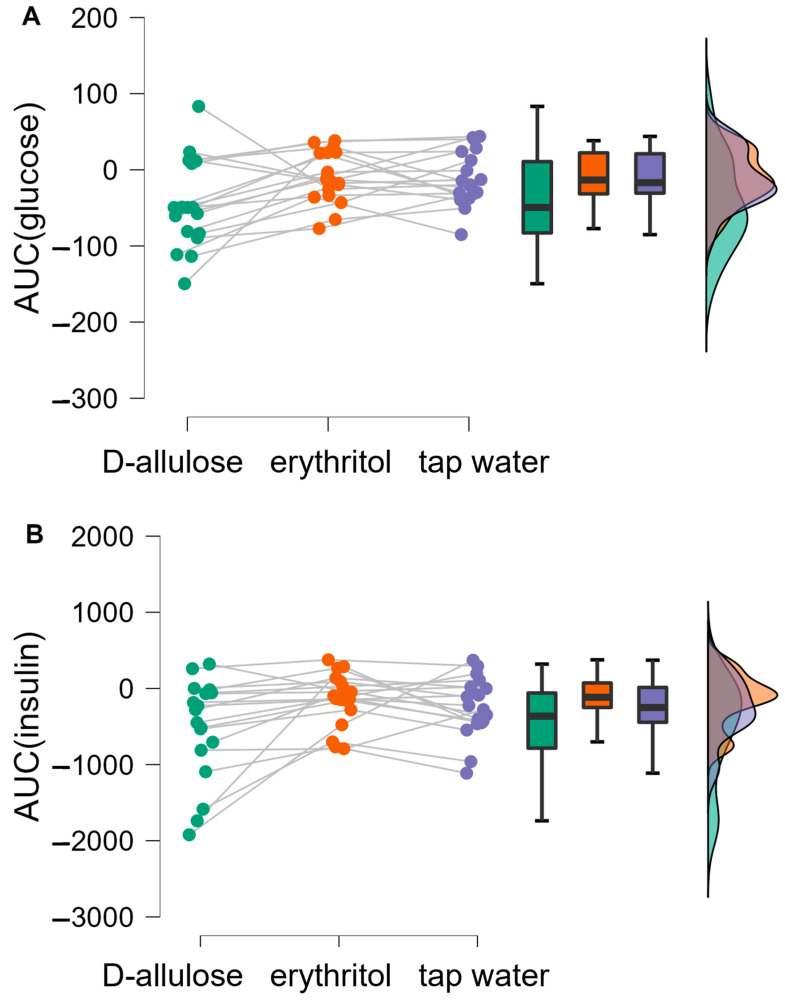
Raincloud plots showing the Bayesian repeated measures ANOVA on the AUC of glucose (**A**) or insulin (**B**) concentrations in response to the intragastric administration of solutions containing 25 g D-allulose (green), 50 g erythritol (orange) or tap water (purple) to 18 healthy subjects.

The Bayesian repeated measures ANOVA on the AUC yielded moderate evidence in favor of a difference between the three solutions in the omnibus test (BF_10_ = 7.50, R² = 0.42 [0.26–0.57]), as well as for the D-allulose vs. tap water post-hoc comparison (BF_10_ = 4.14). Moderate evidence was found in favor of erythritol being no different from tap water (BF_10_ = 0.243) (Figure 3A).

### 3.2. Plasma Insulin

D-allulose decreased plasma insulin, whereas erythritol had no effect compared to tap water (Figure 2B). The main effect of the solution was significant (F (2, 37) = 6.15, *p* = 0.005). The solution-by-time interaction effect was not significant (F (12, 170) = 0.59, *p* = 0.848). Planned contrast analyses show that plasma insulin was lower after D-allulose vs. tap water, but not after erythritol vs. tap water (*p* = 0.005, d_z_ = 0.58 and *p* = 0.320, respectively). The difference between D-allulose and tap water was not confirmed in a Bayesian linear mixed model analysis (0.020 ± 0.755, −95% HPD 1.457–1.495); however, the lack of difference between erythritol and tap water was corroborated (0.027 ± 0.756, 95% HPD −1.444–1.521).

The Bayesian repeated measures ANOVA on the AUC yielded moderate evidence in favour of a difference between the three solutions in the omnibus test (BF_10_ = 4.77, R² = 0.41 [0.25–0.56]), with the evidence for the D-allulose vs. tap water and erythritol vs. tap water post-hoc comparisons being inconclusive (BF_10_ = 1.06 and 0.42, respectively) (Figure 3B).

### 3.3. Plasma Octanoylated Ghrelin

**Figure 4:**
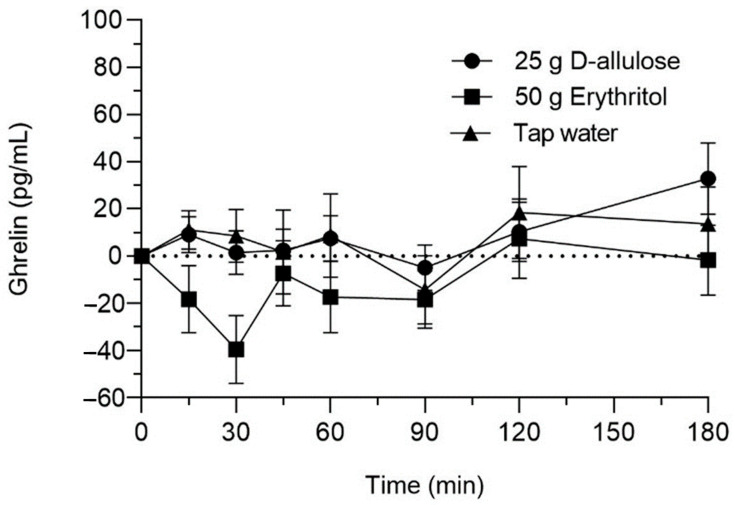
Ghrelin concentrations in response to intragastric administration of solutions containing 25 g D-allulose, 50 g erythritol or tap water to 18 healthy subjects. Data are expressed as mean ± SEM, and changes from baseline values are shown.

D-allulose and erythritol had no effect on ghrelin compared to tap water (Figure 4). Neither the main effect of the solution nor the solution-by-time interaction effect were significant ((F (2, 39) = 2.14, *p* = 0.132) and (F (12, 156) = 0.86, *p* = 0.591), respectively). None of the planned contrast analyses were significant. However, further exploration of the time points at 30 min and 45 min post D-allulose and erythritol administration show a decrease of ghrelin in response to erythritol at 30 min (*p* = 0.026, d_z_ = 0.59), with no effects in response to D-allulose (*p* = 1).

### 3.4. Serum Total Cholesterol

D-allulose and erythritol had no effect on total cholesterol compared to tap water. The main effect of the solution was not significant (F (2, 26) = 0.03, *p* = 0.967). The solution-by-time interaction effect was significant (F (6, 83) = 3.28, *p* = 0.006). None of the planned contrast analyses were significant.

### 3.5. Serum LDL Cholesterol

D-allulose and erythritol had no effect on LDL cholesterol compared to tap water. The main effect of the solution was not significant (F (2, 21) = 0.12, *p* = 0.886). The solution-by-time interaction effect was significant (F (6, 40) = 2.99, *p* = 0.016). None of the planned contrast analyses were significant.

### 3.6. Serum HDL Cholesterol

D-allulose and erythritol had no effect on HDL cholesterol compared to tap water. The main effect of the solution was not significant (F (2, 21) = 0.58, *p* = 0.568). The solution-by-time interaction effect was significant (F (6, 85) = 3.66, *p* = 0.003). None of the planned contrast analyses were significant.

### 3.7. Serum Triglycerides

D-allulose and erythritol had no effect on triglycerides compared to tap water. Neither the main effect of the solution nor the solution-by-time interaction effect were significant ((F (2, 29) = 0.61, *p* = 0.550) and (F (6, 81) = 2.08, *p* = 0.064), respectively). None of the planned contrast analyses were significant.

### 3.8. Serum Uric Acid

D-allulose and erythritol had no effect on uric acid compared to tap water. The main effect of the solution was not significant (F (2, 17) = 0.08, *p* = 0.925). The solution-by-time interaction effect was significant (F (6, 35) = 9.91, *p* = 0.001). None of the planned contrast analyses were significant.

### 3.9. Serum hsCRP

D-allulose and erythritol had no effect on hsCRP compared to tap water. Neither the main effect of the solution nor the solution-by-time interaction effect were significant ((F (2, 23) = 0.51, *p* = 0.606) and (F (6, 83) = 1.21, *p* = 0.309), respectively). None of the planned contrast analyses were significant.

## 4. Discussion

This study aimed to investigate the metabolic effects and safety aspects of the acute intragastric administration of either 25 g D-allulose or 50 g erythritol on glucose, insulin, ghrelin, blood lipid, uric acid and hsCRP concentrations. The results show that: (i) glucose and insulin concentrations did not increase in response to D-allulose and erythritol, compared to tap water; (ii) ghrelin concentrations decreased in response to erythritol (exploratory analysis), but not to D-allulose, compared to tap water; (iii) blood lipids, uric acid and hsCRP were not affected in response to D-allulose and erythritol compared to tap water.

The linear mixed model analysis shows that glucose and insulin concentrations were lower in response to D-allulose, but not erythritol, compared to tap water. However, both Bayesian models did not show evidence of a difference in insulin concentrations in response to D-allulose compared to tap water. The results of D-allulose and erythritol on glucose and insulin concentrations are therefore in line with previous human studies and support the anti-diabetic effects (i.e., no increase in glucose or insulin concentrations) [9,10,11,12,13,22,23,33]. In contrast to the intragastric administration of 25 g of D-allulose given in isolation in our study, these previous studies used oral doses between 2.5–10 g with the addition of either maltodextrin [11], an OGTT [13] or an oral sucrose load [10] assessing post-prandial blood glucose and insulin concentrations. To date, however, the mechanisms underlying the anti-diabetic effects of D-allulose and erythritol are not clear. A study in rats has suggested hepatic glucokinase changes in response to a rare sugar syrup containing D-allulose for 10 weeks as a possible mechanism for the reduction of post-prandial blood glucose [34]. However, it merits further investigation if this mechanism applies to pure and acute D-allulose administration. For erythritol, ameliorated insulin-mediated muscle glucose uptake and reduced intestinal glucose absorption was proposed as a mechanism in diabetic rats [35]. However, chronic intake of erythritol had no effect on intestinal glucose absorption in a recent human study [36]. At least for now, acute ingestion of both natural sweeteners seems to be a helpful alternative compared to sugar, especially for patients with obesity or T2DM.

Anorexigenic and orexigenic hormones play an important role in regulating appetite and satiation. Common mediators are CCK, GLP-1, PYY and ghrelin [37]. Unlike the other hormones, ghrelin is known as a “hunger hormone” and promotes food intake and increases gastric emptying [38,39]. It was shown that ghrelin concentrations were not affected in response to acute or chronic (2 weeks) artificial low-caloric sweeteners such as sucralose or aspartame [25,40,41]. We observed a similar acute effect in this study for D allulose. In contrast the exploratory analysis, erythritol induced a reduction of ghrelin at time point 30 min. The finding for erythritol is in line with the acute pilot study from Sorrentino et al. [25]. Moreover, the results of ghrelin in response to both alternative sweeteners reflect the gastric emptying rates recently reported, with no effect in response to D-allulose and a slowing down in response to erythritol [14]. Thus far, the results from the study in mice by Rakhat et al. [17], where a reduction in ghrelin-responsive neurons in the ARC was reported in response to D-allulose, are not translatable to humans. The sample size of the current study was rather small and further studies are needed to investigate the effects of D-allulose and erythritol on orexigenic hormones.

The potential side effects associated with high sugar intake, especially fructose, are changes in blood lipids, uric acid or hsCRP [1,3].

For both sweeteners, we and others show that D-allulose and erythritol have no clinically relevant effects on blood lipids [18,19,23,33]. Our findings are in line with these studies. However, more long-term studies with D-allulose and erythritol in different patients are needed to investigate the effects on blood lipids on long-term safety.

High uric acid concentrations and inefficient excretion thereof are often associated with hyperuricemia. Besides purine-rich food, other factors such as high fructose or alcohol consumption can trigger this metabolic disease [6,8,42,43]. Since D-allulose is a stereoisomer of fructose, we examined uric acid concentrations. Our finding for acute administration of D-allulose is in line with the study by Hayashi et al. [12] where no effect was found on uric acid during a 12-week period. Of note, the administered dose in the current study was 10 g higher and without the influence of any other nutrients. No effects on uric acid were observed in response to erythritol. This is in line with a recent dose-ranging study where the highest dose of erythritol (50 g) had no effect on uric acid concentrations [23]. However, further studies are needed to test if chronic consumption of D-allulose and erythritol influence uric acid concentrations.

CRP is an acute-phase protein biomarker indicating inflammatory processes in the body [44], whereas the hsCRP is specific to CVD [45]. It was reported that acute and chronic fructose consumption increased hsCRP, possibly leading to systemic inflammation [1,46]. Our results for D-allulose are in line with the study by Tanaka et al. [19] who examined the long-term effects of D-allulose in participants with high LDL cholesterol levels on hsCRP and found no increase during the 48-week trial. To the best of our knowledge, no studies have investigated the effects of erythritol on hsCRP, and in the current study, no acute effect on hsCRP was found. This suggests that the acute administration of both sweeteners does not cause pro-inflammatory effects in the body.

Some limitations need to be considered. First, the design of this acute trial does not allow the investigation of chronic effects of D-allulose and erythritol. Second, additional, at this stage not identified side effects could occur under long-term treatment. Third, the study involved the intragastric administration of two alternative sweeteners by bypassing oro-sensory cues, which may limit translational inferences that can be drawn from the ‘real-life’ consumption of sweeteners (especially over the longer term). Fourth, a comparison of D-allulose and erythritol to a sucrose solution would be informative.

In conclusion, this study shows that the acute intragastric administration of the two alternative sweeteners D-allulose and erythritol, has beneficial physiological effects regarding glycemic control and ghrelin, and exhibits a clinically favorable safety profile with respect to blood lipids, uric acid and systemic inflammation. This combination of properties identifies D-allulose and erythritol as excellent candidates for effective and safe sugar alternatives.

## Data Availability

The data presented in this study are available on https://github.com/labgas/proj_erythritol_1.
